# How and why adults use cannabis during physical activity

**DOI:** 10.1186/s42238-022-00134-z

**Published:** 2022-05-18

**Authors:** Whitney L. Ogle, Gregg J. Gold, Lukas E. Coppen, Claire Copriviza

**Affiliations:** 1 Department of Kinesiology & Recreation Administration, Cal Poly - Humboldt, 1 Harpst St., Arcata, CA 95521 USA; 2 Humboldt Institute for Interdisciplinary Marijuana Research, 1 Harpst St., Arcata, CA 95521 USA; 3 Department of Psychology, Cal Poly - Humboldt, 1 Harpst St., Arcata, CA 95521 USA

**Keywords:** Cannabis, Exercise, Drug use in sport, Physical activity

## Abstract

**Background:**

With increased legalization of cannabis, users are combining cannabis with exercise. The purpose of this study is to understand how and why people use cannabis when participating in physical activity.

**Methods:**

A convenience sample of cannabis users participated in an anonymous online survey created by the authors regarding exercise habits while under the influence of cannabis, perceived benefits, unanticipated experiences related to cannabis and exercise, and demographics.

**Results:**

One hundred thirty-one respondents (18–55 years) were eligible and began the survey. Nearly 60 physical activities under the influence of cannabis were reported. The most frequently reported modes of exercise included hiking (60%), yoga (58%), and aerobic machines (50%). The primary reasons for using cannabis before exercise were “helping to focus/concentrate” (66%), “helping enjoy the exercise experience” (65%), and “enhancing mind-body-spirit connection” (65%). Thirty-three participants reported “yes” or “maybe” regarding having an experience they “didn’t anticipate or desire while exercising under the influence of cannabis.”

**Discussion:**

Participants’ reasons for exercising with cannabis span the physiological, psychological, neuromotor, and even spiritual domains. However, some reported an undesired experience when pairing cannabis with exercise. While this was an online survey with a small sample size, our results agree with and contribute to the growing research investigating cannabis use and physical activity participation.

**Conclusion:**

We found considerable heterogeneity in types of physical activity participation under the influence of cannabis, as well as perceived benefits of use. This study provides directions to further explore the risks and benefits of combining cannabis with physical activity.

## How and why adults use cannabis during physical activity

Cannabis is one of the most widely used “illicit” drugs (Center for Behavioral Health Statistics and Quality (CBHSQ) 2016), and ranks second highest in reported drug consumption among athletes (Brisola-Santos et al. 2016). While research investigating the impacts of cannabis use on exercise performance began over 50 years ago (Maksud and Baron 1980; Steadward and Singh 1975; Kvålseth 1977; Renaud and Cormier 1986; Renault et al. 1971; Peters et al. 1976; Manno et al. 1970; Tashkin et al. 1973), the legal status of cannabis has made this research difficult to perform (Piomelli et al. 2019). At this time, controlled laboratory studies such as those by Renaud and Cormier 1986, Steadward and Singh 1975, and Tashkin et al. 1973 from the 1970s and 1980s are the exception rather than the norm when investigating cannabis and exercise. Even in the original laboratory research on the effects of cannabis on exercise performance, the cannabis potencies used do not reflect what is currently commercially available, making their findings hard to extrapolate to present day (Gillman et al. 2015; Vergara et al. 2017; Bidwell et al. 2018).

In the absence of lab-based research, there has been an increase in anecdotes in popular media advocating cannabis use in conjunction with exercise coming from recreational, current, and retired professional athletes (Armstrong 2018; Borchardt 2016; Brand 2019; deBara 2019; Lev 2020; NORML 2015; The Athletics Issue 2018; Nguyen 2019). Because the plural of anecdote is not data, it is clear that there is a growing need to better understand the relationship between cannabis use and physical activity participation (Nguyen 2019; Docter et al. 2020; Kramer et al. 2020).

Recent research has aimed to elucidate this relationship through survey studies of cannabis users who report using cannabis before or after exercise (YorkWilliams et al. 2019; Zeiger et al. 2019; Lisano et al. 2019). For example, YorkWilliams et al. 2019 found that not only did respondents who used cannabis before exercise report increased levels of physical activity participation, but doing so increased their enjoyment of exercise and motivation to exercise. In a survey of athletes, Zeiger et al. 2019 found higher positive subjective experiences such as well-being and calm than adverse experiences with cannabis. Lisano et al. 2019 survey of people who use cannabis before, during, or after exercise revealed more insight about the perceived benefits, consumption patterns, and exercise habits in this population, where people who used cannabis prior to exercise reported positive effects on their performance. In addition, they found “pain management” as a reason for people to use cannabis at all time points (before, during, and after) relative to physical activity. These findings are in line with previous reviews of the potential uses of cannabis as it relates to perceptions of exercise performance, motivation, and recovery from exercise (Gillman et al. 2015).

Building on the work of YorkWilliams et al. (2019), Zeiger et al. (2019), and Lisano et al. (2019), the purpose of this study was to further investigate the uses of cannabis and physical activity to gain a more complete understanding of how and why the public is using cannabis with exercise. Through a survey of people who reported their experiences using cannabis with physical activity, our research adds to small body of recent existing literature, and intends to provide a base for future research.

## Method

Participants were recruited to complete an online survey between April and October of 2018, via a convenience sample of people who saw our social media posts, flyers in Arcata California, or through word of mouth. Each of the recruitment materials provided a link to questionnaire hosted online by Survey Monkey. After reading a description of the study, participants provided anonymous consent by agreeing to participate in the study on the first page of the Survey Monkey link, clicking “yes”, which allowed them to access the survey. If they did not consent to participate, clicking “no”, they were unable to access the rest of the survey. Participants were invited to participate if they were self-reported to be over the age of 18, a recreational cannabis user, and if they participated in physical activity outside of activities of daily living. Based on their response to the first question, “In a typical week, how often do you use cannabis or cannabinoid products before or during exercise?”, if a participant chose 25% of the time or greater, they continued with the rest of the questions about cannabis use and exercise. If they responded rarely or never (*n* = 60), they were excluded from the present study, as we were only interested in participants who combined cannabis with exercise. The study was approved through the Institutional Review Board at Humboldt State University. To ensure anonymity, no data was collected that could reveal the identity of a participant. Sample size was limited by the time available to collect the convenience sample, and no data was subsequently collected. Thus, sample size was fixed before any data analysis began.

### Instrument

At the time of this survey’s creation, no existing questionnaire was deemed suitable, so one was created based on anecdotal reports from secondary sources (blogs, social media, magazine articles, for example (Armstrong 2018; Borchardt 2016; NORML 2015; The Athletics Issue 2018). The survey included 32 questions including multiple choice, Likert scale, and check all that apply questions in 5 sections. Participants were asked about their use of cannabis with physical activity (17 questions; e.g., timing, methods of cannabis administration, and types of physical activities), general cannabis use (4 questions; e.g., frequency, and history of cannabis use), general physical activity participation (1 question; history of physical activity participation), unanticipated experiences (5 questions; e.g., product used, method of administration, and other substances used), and demographics. Time to complete the survey was between 5 and 10 min. Participants could choose a response, write in a response, or skip questions.

### Data analysis

Data was downloaded from SurveyMonkey and analyzed using SPSS 26. Before analysis, data was analyzed for outliers and checked to make sure it met statistical assumptions for analysis. Response frequencies and percentages (rounded to the nearest whole number) were determined. For some questions, percentages add up to over 100%, as participants were not restricted to choosing just one answer. The potential relationships between categories of interest like age or gender were investigated using ANOVA, *t* tests, or chi-square analysis as appropriate.

## Results

Two hundred eight participants began the survey, one was disqualified for not indicating they had read and agreed to the informed consent page. Of the remaining, 191 completed question two regarding cannabis use and exercise. Our criteria for analysis was that participants indicated they used cannabis in conjunction with exercise at least 25% of the time. Although 131 indicated they used at least 25% of the time, 30 participants skipped almost all of the subsequent questions. Thus, 101 was the most common number of participants who met the criteria, and whose responses were therefore available for subsequent analysis.

### Participants

Of those who met the criteria for the survey and completed the demographic section at the end of the survey (*n* = 81), there were 40 males, 40 females, and 1 non-binary participant. Seventy-nine percent of participants (*n* = 80) were between the ages of 18 and 34, and 21% between the ages of 35–55. Most participants (*n* = 81), identified themselves as healthy or very healthy (69%), and 30% perceived their health as neutral. Forty percent of participants (*n* = 81), identified as being a college graduate, with 27% reporting some college and 14% a Master’s or equivalent. When asked how many years they had been consuming cannabis or cannabinoid products (*n* = 82), 74% reported five or more years, 21% 3 to 5 years, and 5% 2 years or less. Regarding frequency of use, 79% indicated daily and 21% indicated weekly. Comparing their general cannabis consumption to use prior to exercise (*n* = 100), 10% said more, 55% about the same, 30% less, and 5% significantly less. When asked “how many times per day do you typically use cannabis”, 50% reported three to five times a day or more, and 50% reported zero to two times a day.

Participants (*n* = 101) were asked about their skill level in the activities that they participated in. The majority identified their skill level as intermediate (41%) or advanced (50%), with the remaining 10% split between novice and professional (4% and 6% respectively). When asked about their length of cannabis use with physical activity, 57% indicated five or more years, 21% 3 to 5 years, and 22% for 2 years or less. When compared to exercise in general, 64% said they had been participating in regular exercise for more than 5 years, 20% 3 to 5 years, and 16% 0 to 2 years.

Participants who were included in the study (see above), were asked about how often in a typical week they used cannabis with exercise (*n* = 131). The choices were 100%, 75%, 50%, and 25%. Forty-one percent chose 100% of the time, 23% chose 75% of the time, 25% chose 50% of the time, and 11% of participants chose 25% of the time. When asked about the timing of cannabis or cannabinoid product use during physical activity (*n* = 100), 87% said they used cannabis shortly before exercise, 55% shortly after, 43% hours after, 31% hours before, and 26% during exercise. To see if there might be a significant relationship between how often in a typical week participants indicated they exercised with cannabis, and how many years they had been using with exercise, a chi-square analysis was performed. No significant relationship was found, *χ*^2^ (6, *N* = 101) 10.58, *p* = .10.

### Cannabis and exercise

The most popular method of consuming cannabis before exercise (*n* = 101) was smoking flower (53%) via water pipe (43%), bowl (45%), or joint (35%). Subsequent popular methods were vaporizers (38%), extracts (wax, shatter, dabs, etc.) totaling 33%, edibles such as candy bars, drinks, and tinctures (23%), and topicals (12%). Participants indicated which strains of cannabis they believed they used as follows: sativa dominant (64%); hybrid (46%); Indica dominant (25.7%); non-psychoactive cannabidiol (CBD) products (17.8%); unknown (15.8%); and other (5.9%).

Respondents reported having participated in 54 types of physical activity under the influence of cannabis (see Table 1). The top 5 most common modes of exercise while using cannabis were hiking (60%), yoga (58%), aerobic machines (51%), weightlifting (44%), and walking (43%).

**Table 1:** Percent of participants reporting cannabis use by activity^a^

Percentage (*n* = 101)	Activities are in order of highest percentage within each row
60 to 50%	Hiking; yoga; aerobic machines
49 to 40%	Weight lifting; walking
39 to 30%	No activities were in this range
29 to 20%	Swimming-indoor pool; running-sprint/mid distance; running- long distance; cycle-road; kayak/canoe; rock climbing; snowboarding; swimming-open water
19 to 10%	Dance; skiing; cycle-mountain bike; disc golf; skateboarding; golf; stand up paddle boarding; pilates; bowling; basketball; surfing
9 to 1%	Volleyball; baseball/softball; slacklining; other; shooting; soccer; tennis; ultimate frisbee; boxing; gymnastics; racquetball; barre; football; kickball; pole dancing; water polo; circus; sailing; martial arts; rowing; water skiing/wakeboarding; wrestling; archery; equestrian; field events; hockey; caving; cheerleading; kiteboarding; quidditch

^a^Multiple distinct choices were possible

Taking a closer look at the top five most preferred types of exercise performed under the influence of cannabis (hiking, yoga, aerobic machines, weightlifting, and walking), the preference for strain of cannabis was relatively consistent. With *n* = 101, between 37% and 41% of participants said they had used sativa while engaging in hiking, yoga, aerobic machines, or weightlifting, and 26% while walking. For hybrid use, numbers ranged from 25 to 30% of participants among all activities, with Indica and CBD in the 6 to 15% range. Among those surveyed, sativa and hybrid strains were the most commonly used in the most popular exercise activities.

With respect to the most five preferred types of exercise under the influence of cannabis, among those who indicated their gender identity, significantly more females walked (27%) than males (14%), with *χ*^*2*^ (2, *N* = 81) 7.61, *p* = .022. Also, significantly more females practiced yoga (35%) than males (22%), with *χ*^2^ (2, *N* = 81) 6.42, *p* = .040. There were no significant gender differences for hiking, aerobic machines, or weightlifting.

After using cannabis, participants (*n* = 99) reported they exercised outside (74%), at home (59%), in the gym (51%), and 7% other. When under the influence of cannabis or cannabinoid products, 60% reported exercising individually, 14% in a group setting, and 40% reported participating in both individual and group contexts.

There were a variety of reasons (Table 2) participants (*n* = 100) indicated they consumed cannabis prior to physical activity. The top 5 reasons included helping with focus/concentration (67%), helping enjoy exercise (66%), enhancing mind-body-spirit connection (65%), staying in the zone (62%), and enhancing body awareness (53%). While participants reported using cannabis with exercise for seemingly contradictory reasons, such as increasing body awareness and decreasing body awareness, there were more respondents who claimed increases in awareness and focus. A greater percentage of the sample reported using cannabis with exercise to manage psychological and psychosocial aspects of exercise, such as sense of time, enjoyment, and self-consciousness, than to manage pain or to increase performance.

**Table 2:** Percent of participants reporting why they used cannabis while exercising^a^

Percentage reporting (*n* = 100)	Activities are in order of highest preference within each row
69 to 60	Helps me focus/concentrate; helps me enjoy the exercise experience; enhances mind/body/spirit connection; keeps me in the zone
59 to 50	Enhances body awareness; to relieve muscle tension
49 to 40	To be more observant/aware; improve flexibility; to decrease inflammation
39 to 30	To decrease my sense of time during exercise; as a reward for exercising; to be more conscious of my breath during exercise; helps sleep; recovery from pain; as ritualistic medicine; increase energy; to manage pain in order to tolerate exercise; enhance physical performance; to manage social anxiety
29 to 20	Improve patience from frustration; to improve creativity during exercise; my friends do it/we do it together; delays onset of muscle soreness; to regulate my breathing
19 to 10	Distraction from exercise; to decrease self-consciousness; to regulate my heart rate
9 to 1	To be less observant/aware; decreases body awareness; as a neuroprotectant; Other: helps me achieve that runners high sooner; relieve stomach pain; cause my life is dope and I do dope shit; helps control my blood sugar; I am diabetic; relaxes me and puts me in a calm creative headspace

^a^Multiple distinct choices were possible

Table 2 shows why participants used cannabis in conjunction with exercise with multiple distinct choices possible (*n* = 100).

Participants were asked to compare how they felt about exercising with cannabis compared to exercising without cannabis on a 1–5 scale from strongly disagree to strongly agree. There were four categories, and in every category the majority indicated they felt more positively about their experiences when exercising with cannabis as shown in Table 3. There were no significant differences in how participants felt when comparing within categories by age or gender (*p* > 0.31).

**Table 3:** How participants indicated they felt when comparing exercise with cannabis to exercise without on a 1–5 scale with 5 = strongly agree and 1 = strongly disagree (*n* = 101)

	Strongly agree	Agree	Neutral	Disagree	Strongly disagree	Mean and standard deviation
More focused	29%	43%	19%	8%	1%	*M =* 3.92, SD = .95
More satisfied	32%	37%	26%	5%	1%	*M* *=* 3.93, SD = .92
More productive	18%	44%	29%	9%	1%	*M* = 3.68, SD = .90
Better form	19%	34%	39%	8%	1%	*M* = 3.61, SD = .92

When asked if they had ever had an experience they “didn’t anticipate or desire while exercising under the influence of cannabis” (*n* = 80), 25% said yes, 13% said maybe, and 63% said no. Participants (*n* = 33) could choose from a list of unanticipated experiences that might apply with multiple choices possible, or could write a response. The most chosen experiences were “getting too high to be effective at exercise” chosen 17 times, followed by “heart racing” 16 times, and “lightheadedness” 13 times. See Table 4.

**Table 4:** Unanticipated/undesired experiences while exercising with cannabis^a^

Number of times chosen (*n* = 33)	Unanticipated/undesired experience
17	Got too high to be effective at exercise
16	Heart racing
13	Lightheadedness
12	Paranoia
11	Anxiety
9	Weakness
6	Loss of coordination
5	Difficulty breathing/asthma; poor reaction time
4	Passed out; hallucinations; loss of balance
2	More risky behaviors; enhanced pain perception
1	Injury; other: anxiety of people in gym; too sleepy; dizziness, nausea; felt dehydrated; laughing uncontrollably

^a^Multiple distinct choices were possible

To test if there was a relationship between the length of time a participant had been consuming cannabis and whether they had an unanticipated or undesired experience a chi-square analysis was performed, and no significant relationship was found with *χ*^*2*^ (4, *N* = 80) 1.78, *p* = .77. Additionally, to see if there could be a relationship between gender and if they had an unanticipated or undesired experience, a chi square analysis was performed, again, no significant relationship was found with *χ*^*2*^ (4, *N* = 79) 1.98, *p* = .74.

When asked for more detail about the unanticipated experience (*N* = 32), 15 said they did not know the strain, 7 chose Indica, 6 chose sativa, 4 chose hybrid, and no-one chose CBD. The most commonly chosen form of administration (with multiple choices possible) regarding that experience was smoking flower (19), edibles (12), and dabs (9).

Participants were also asked, “were you taking any other substances that may have contributed to this experience.” Of the 25 participants who answered this question, 10 reported caffeine, 7 reported other, 4 reported pre-workout supplement, and 2 prescription medication. To see if there was a relationship between other substances and unanticipated experiences, a chi-square was performed, but no significant relationship was found with *χ*^*2*^ (8, *N* = 25) 6.10, *p* = .636.

## Discussion

In this study, we identified characteristics of adults who use cannabis prior to and during exercise, including types of cannabis products used, types of physical activities engaged in under the influence, where and when people are using, and their motives for combining cannabis with exercise. We found considerable heterogeneity in types of physical activity participation under the influence of cannabis, as well as perceived benefits of use. The focus of this discussion will be on how we can use the information gathered from the current survey to inform future research directions in the field of cannabis and exercise science.

The respondents of this study were mostly heavy chronic cannabis users, reporting daily use for over 5 years. Of the respondents who completed the demographics section of the survey, we found an equal number of male and female participants with one identifying as non-binary. This finding is similar to that of Lisano et al. (2019), who also found an equal number of male and female participants, but in contrast to YorkWilliams et al. (2019) and Zeiger et al. (2019), who reported a greater percentage of male participants in their survey. With regard to the top 5 activities people participated in under the influence of cannabis, females paired cannabis with walking and with yoga significantly more than male participants, but there were no other significant gendered differences (such as unanticipated experiences, or how they felt about exercise under the influence). Future research could continue to investigate the role that gender identity may play in the use of cannabis with exercise, particularly in the transgender and non-binary communities where there are more barriers to physical activity participation (Jones et al. 2017).

The most common forms of physical activity under the influence of cannabis reported in our study included: hiking, yoga, aerobic machines, walking, and weight lifting. These activities are fairly representative of the common forms of exercise in Americans (Bureau of Labor Statistics, U.S. Department of Labor 2016), though in our sample we had a higher frequency of participants who reported yoga, and a lower frequency of running and swimming. However, these findings are similar to those from Lisano et al. (2019), where the most frequent activities reported in people who use cannabis before exercise were hiking, running, yoga, cycling, and resistance training, and those who used cannabis during physical activity were hiking, golf, yoga, and skiing/snowboarding. The slight differences between our findings and those from Lisano et al. (2019) may be related to activities that are common and accessible based on geographic location.

Something to consider moving forward is that the relative ratio of cannabis users may be different depending on the sport or activity. For example, while hiking was the most common activity cited in our study, we do not expect that the majority of people hiking are incorporating cannabis into their hikes. Previous research has found that cannabis use was more common in adolescents who participated in more extreme “sliding” sports such as windsurfing, skiing, snowboarding, and surfing (Lorente et al. 2005). The relative percentage of cannabis users within each sport/activity is likely different, and may reveal an interesting link between cannabis and the psychosocial, neuromotor, and physiological domains of sport.

Ultimately, we found that people engage in a variety of physical activities under the influence of cannabis, which is in line with previous research (Lisano et al. 2019). With nearly 60 physical activities reported in our sample, there were a variety of activities that require different energy systems, levels of concentration, power outputs, levels of competitiveness, precision, accuracy, time, and a mix of team and individual sports. This finding opens the door to an almost limitless supply of research questions for future investigations regarding how cannabis may impact exercise performance. For example, of the six most common forms of physical activity, five would be considered more aerobic activities, with weight lifting typically considered more anaerobic. Sparling et al. (2003) first discovered that moderate intensity aerobic exercise stimulates the endocannabinoid system, leading to the “runner’s high” which may increase the motivation to exercise. Perhaps consuming cannabis prior to a workout may stimulate the endocannabinoid system so that the user feels the effects of the “runner’s high” earlier in the workout. Participants could also be using cannabis to increase the sense of reward of an activity they are participating in at a lower intensity level, or an anaerobic activity that has not been found to activate this system. In future investigations, it will be important to critically evaluate the timing, dosage, method of consumption, and their effects on both aerobic and anaerobic performance (Ogle, 2021).

The majority of our sample reported intermediate or advanced skill levels in the activities they were engaging in while co-using. When considering the most common modes of physical activity reported (i.e., hiking, yoga, aerobic machines, walking), an intermediate or advanced skill level is appropriate. A small percent of the sample reported being a novice, potentially indicating that individuals were not learning new activities under the influence of cannabis. Previous research has shown lower levels of plasma brain-derived neurotrophic factor (BDNF) in physically active cannabis users compared to nonusers (Lisano et al. 2020). Because of BDNF’s role in neuroplasticity and motor learning (Fritsch et al. 2010), further investigations on the impact of cannabis on motor learning is warranted. Also, a small percent of the sample reported professional status in the sport that they participated in, similar to Zeiger et al. (2019). This small number of professional athletes in our sample of those who exercised with cannabis could be a reflection of the small number of professional athletes compared to the general population. However, it also may be related to regular drug testing in some professional sports, which would discourage professional athletes from using cannabis (for more information on cannabis prohibition in sport see Huestis et al. (2011) or Ogle (2021).

Only a small portion of our sample reported not knowing the cannabis strain consumed prior to physical activity, suggesting our sample may have had an educated approach towards consumption before exercise. In addition, the majority of participants reported using Sativa-dominant strains, typically associated with more of an energizing effect (Backes 2017) so it is reasonable that more people would prefer to use sativa-strains prior to exercise. This finding agrees with previous research, where participants reported greater use of Sativa products before physical activity, and Indica products after physical activity (Lisano et al. 2019). While a terpene profile (organic compounds that provide aroma and flavor in cannabis), may be more helpful in identifying the effects of cannabis on humans (Piomelli and Russo 2016), at this time cannabis products are typically marketed as Indica-dominant, sativa-dominant, or hybrid. Future research on cannabis and exercise should carefully consider these profiles, especially when evaluating the effects on human performance.

For participants who reported unintended experiences, most reported that they did not know the strains they were using. Based on the analyses performed, there also did not appear to be a relationship between gender or length of time participants had been using and unanticipated experiences. It is important to note that there were zero cases of negative experiences when people reported using non-psychoactive CBD products prior to exercise. The consumption type that led to the unanticipated experience was primarily smoking flower, edibles, and dabs. This is interesting because edibles were not a common method of administration overall in this study, but produced a relatively large percentage of the negative experiences. Research has found that people who use edibles have greater odds of an unexpected high (Allen et al. 2017), which could be related to unintended overconsumption or due to label inaccuracies (Vandrey et al. 2015).

We found that the majority of our sample reported smoking cannabis (bowl, joint, or bong), followed by vaporizing, extracts, edibles, and topicals. Survey data has shown that smoking continues to be the most common method of THC administration (Hazekamp et al. 2013; Knapp et al. 2019), and this finding supports the findings of Lisano et al. (2019) who also identified that inhalation was the most common method of administration before, during, and after physical activity. Each of these methods of administration are worth investigating further as it relates to exercise performance. Edibles take longer to metabolize, so the effects take longer to take effect, and last longer as compared to smoking (Allen et al. 2017). Therefore, future investigations should consider time of ingestion when looking at the effects of different modes of ingestion on exercise function, similar to the design by Renault et al. (1971).

Participants reported using cannabis when exercising outside, and typically use cannabis shortly before exercise: using either at home, outside or at the gym. Logistically, this means that some people may be using at home, and then driving to exercise locations. Cannabis consumption prior to driving presents a safety risk, particularly with those who are only occasional users (Hartman and Huestis 2013). While a further investigation into this is outside the scope of the current study, it is worth contextualizing the reality of using cannabis before exercise.

With our sample, the most common reasons for using cannabis in conjunction with exercise included: “helped me focus/concentrate,” “helps me enjoy the exercise experience,” “enhances mind/body/spirit connection,” and “keeps me in the zone.” Helping with focus/concentration is similar to “staying in the zone” in terms of concentrating one’s mind on a task at hand and shutting out distractions. Helping enjoy exercise may indicate that cannabis increased the reward value of activity, while enhancing mind-body-spirit connection may indicate that cannabis may increase the reward, but in more than just purely the physical realm. Enhancing body awareness may indicate that cannabis allows the participant a better understanding of how their body is responding to the activity. These results are similar to, but do not quite match the reasons for cannabis use before, during, or after physical activity from those of Lisano et al. (2019). They found pain management/relief, improving focus, and relaxation were the most reported reasons for use of cannabis before exercise, with to increase energy, improve enjoyment of activity, and pain management as the most common reasons to use cannabis during physical activity. This could be related to the number of options available to participants in the present study as compared to the survey by Lisano et al. (2019), where respondents provided open-ended answers to questions about reasons for use of cannabis before, during, and after physical activity, which was then coded and presented in their results.

The majority of our sample agreed that they felt more satisfied, focused, and productive during their workouts. These results support YorkWilliams et al. (2019) where cannabis increased enjoyment of, and motivation to exercise. We did not find as high percentages of participants claiming use of cannabis to aid in recovery as YorkWilliams et al. (2019) but this may be because we were specifically interested in only people who used before or during exercise, rather than those who use cannabis after exercise.

The present study expanded upon and provided more in-depth choices than previous studies in order to gain a more complete picture of the myriad of reasons why adults would combine cannabis and exercise. Participants’ reasons for exercising with cannabis span the physiological, psychological, neuromotor, and even spiritual domains. What is not clear yet is whether these perceived benefits of using cannabis prior to exercise actually lead to measurable changes in exercise performance. Older cannabis and exercise research found mixed results about the effects of cannabis on different aspects of performance (Maksud and Baron 1980; Steadward and Singh 1975; Kvålseth 1977; Renaud and Cormier 1986; Renault et al. 1971; Peters et al. 1976; Manno et al. 1970; Tashkin et al. 1973), but the strains used in the studies do not reflect the strains that are commercially available today (Vergara et al. 2017; Bidwell et al. 2018), and therefore need to be reassessed to determine the actual impact on performance. Our expanded list of reasons why adults use cannabis with exercise should guide future research endeavors based on the experiences of those who combine the two. Of particular interest, future research should carefully consider how to assess changes in focus/concentration, and body awareness when pairing cannabis with exercise, and how that may impact performance.

While our participants felt they personally benefited from pairing cannabis and exercise, it may not be beneficial to all. About one third of our participants reported having an unanticipated or undesired experience while using cannabis in conjunction with physical activity. Yet, 78% of the participants in this study had been exercising with cannabis for over 3 years making them experienced users, and no significant relationship was found between years of use and unanticipated experiences. Previous research has found that the effects of cannabis on different aspects of performance may differ between long-term and short-term cannabis users (Kirk and de Wit 1999; OʼLeary et al. 2003; Solowij 2002), so the impact of length of use should be considered in future investigations of cannabis and human performance.

The most common unanticipated experiences include heart racing, got too high to be effective at exercise, anxiety, paranoia, and lightheadedness. Cannabis has been shown to be a bronchodilator, and increases heart rate immediately after use (Renault et al. 1971; Kennedy 2017), so the experience of “heart racing” may be related to an individual’s physiological response to cannabis consumption, or could also be due to psychological responses like anxiety or paranoia. Anxiety, paranoia, and lightheadedness might be related to dosage, and will need to be carefully considered in future lab-based studies. The finding of “got too high to be effective at exercise” feeds into the “lazy stoner” archetype, but is likely related to dosage. Dietrich and McDaniel (2004) noted that people may experience a biphasic response to cannabis where, depending on the dosage, can elicit either motor inhibition or excitation. If the goal is to exercise under the influence of cannabis, we assume that participants could adjust their cannabis dosage to ensure they are not overconsuming so that they can participate as intended. Future research and educational outreach should consider the harm reduction and benefit maximization for those who decide to combine cannabis use with exercise.

### Limitations

The primary limitation of the study was that it was a subjective survey where recall bias may have influenced the results collected from a relatively small, self-selected convenience sample, and therefore may not be representative of all people who use cannabis with exercise. The survey was online and we did not include a question asking the respondents’ geographic location, so it is unknown whether all the participants were in the USA and, if so, whether they were in a state with commercial cannabis sales. In states where cannabis is legal for medicinal or recreational sales, people are much more likely to know exactly what product they are consuming. In addition, only respondents who reported an unanticipated or undesired experience with cannabis and exercise were asked a question about concurrent use of other substances. Some cannabis users or people participating in physical activity may use other substances at the same time such as caffeine or supplements, future investigations should consider the role that concurrent substance use may play in modulating physical activity performance.

The survey we created was based on anecdotal accounts of cannabis use and exercise from popular media anecdotes because at the time, there was not an established or validated survey of cannabis use and exercise. While our survey was not validated, it was piloted with a small group of people who use cannabis with exercise prior to launching recruitment. The purpose of this survey was to explore the experiences and perspectives of people who use cannabis with exercise in order to highlight factors worthy of future investigation in the field, and not to validate a survey. Because the options for responses to the question, “Why do you use cannabis in conjunction with exercise (check all that apply)” were based on anecdotes from popular media, some of the responses such as “enhances mind/body/spirit” connection may be harder to investigate further since the field of kinesiology does not typically use such language. Nonetheless, the subjective experiences and interpretation of the language used to describe their experiences are worthwhile to consider in preparation for lab-based studies of cannabis and kinesiology that are guided by experience and relevant to the public. We hope that this study will set a precedent for future experimental designs in the fields of exercise science, sport psychology, and motor learning and control with respect to cannabis once restrictions are lifted.

## Conclusion

This study found that people are using cannabis in conjunction with exercise for a wide variety of perceived benefits among a remarkably diverse set of physical activities. Though about one third of the sample had unexpected experiences, judging from the length of time and percentage of time our sample has combined cannabis with exercise, it is reasonable to assume participants judge the benefits were/are worth it. As the use of cannabis in general becomes more normalized among the population, how and why adults choose to use cannabis in combination with exercise will become increasingly important to guide future research and educational campaigns. Future research should build on the results of surveys such as this to investigate how cannabis use before exercise actually modulates physical activity participation and exercise performance in order to accurately inform the general public of specific risks and benefits. Investigating the impact of cannabis on different forms of exercise will reveal valuable links into the psychosocial, neuromotor, and cardiopulmonary effects of cannabis on aspects of sport and exercise performance*.*

## Data Availability

The datasets used and analyzed during the current study are available from the corresponding author on reasonable request.
